# Brain spatial reconciliation through multisensory integration in the impact of pandemic fatigue on workplace

**DOI:** 10.3389/fnhum.2024.1419889

**Published:** 2024-12-06

**Authors:** Rizka Tri Arinta, Prasasto Satwiko, Robert Rianto Widjaja, Sri Kusrohmaniah

**Affiliations:** ^1^Doctoral Program in Architecture, Faculty of Architecture and Design, Soegijapranata Catholic University, Semarang, Indonesia; ^2^Department of Architecture, Faculty of Engineering, Universitas 17 Agustus 1945, Semarang, Indonesia; ^3^Department of Architecture, Faculty of Engineering, Universitas Atmajaya, Yogyakarta, Indonesia; ^4^Department of Architecture, Faculty of Architecture and Design, Soegijapranata Catholic University, Semarang, Indonesia; ^5^Faculty of Psychology, Universitas Gadjah Mada, Yogyakarta, Indonesia

**Keywords:** brain spatial reconciliation, multisensory integration, workplace, Bayesian brain, pandemic fatigue

## Abstract

The COVID-19 pandemic has highlighted the prevalence of fatigue, reduced interpersonal interaction, and heightened stress in work environments. The intersection of neuroscience and architecture underscores how intricate spatial perceptions are shaped by multisensory stimuli, profoundly influencing workers’ wellbeing. In this study, EEG and VR technologies, specifically the *Emotiv Epoc X*, were employed to gather data on perception and cognition. Through the analysis of statistical data, *independent component* analysis (ICA), and perception metrics, the research explored the brain’s responses to various sensory stimuli encountered in the workplace. This research aims to examine how individuals adapt to work environments that expose them to multiple sensory stimuli, by observing brain activity and perception processing. The findings indicate that integrating multisensory stimuli, such as light, sound, and smell, can significantly enhance employees’ performance and perception of their workspaces. *The Bayesian brain* mechanism, which prioritizes key sensorimotor inputs, plays a critical role in continuously adjusting the brain’s perception of sensory information. This mechanism operates through sensory weighting, wherein the brain assigns greater importance to the most relevant sensory inputs, depending on the specific demands of the work environment. For instance, visual elements, such as lighting and color schemes, along with olfactory stimuli in high-density environments, are instrumental in shaping workers’ perceptions of the spatial dimensions, ambiance, and emotional responses within the workplace. This underscores the potential of multisensory integration as a form of reconciliation between architecture and the cognitive demands of office spaces.

## 1 Introduction

The COVID-19 pandemic has left a lasting effect on human reconciliations, especially concerning fatigue due to strict health protocols, confined communication and interplay, and frequent publicity of distressing information. This enjoyment has had an extended effect even after the pandemic ended. People have become extra touchy in poorly lit areas (Malnar and Vodvarka, 2004; Spence, 2020), coughing and sneezing sounds (Michalak et al., 2020), and the smell of disinfectants (Allen, 2023). This perception of fatigue shapes the spatial reports stored in reminiscence and can result in terrible perceptions of day-to-day lifestyles, specifically for indoor people. Stressors within the work surroundings, together with high workload, isolation, long working hours, position conflicts, lack of autonomy, profession development limitations, complex relationships with colleagues and executives, bullying, harassment, and poor organizational climate, contribute to administrative center strain (Sander et al., 2019).

The development of workspace design during the pandemic has highlighted the need for effective and responsible use of color to foster communication and collaboration with others (SMOW, 2021). In the case of public provider workplaces, particularly Population Data Recording Places (PCAO Disdukcapil) in Indonesia, these offices have become critical facilities for population administrations, significantly for recording the sizeable boom in loss of life records throughout the pandemic. This registration method nevertheless requires direct contact between people and record applicants. Although local governments have tried to shift the online application, Semarang’s citizens mostly have struggled to conform during the pandemic. As a result, the Disdukcapil workplace needs to facilitate direct contact while minimizing the number of candidates. This phenomenon is not particular to the Disdukcapil workplace but is also common in many authorities’ offices that manage public services. This observation bureaucracy is the premise for studies and design development, focusing on personalization that developed for the pandemic, warranting further research, especially thinking about improvements in neuroarchitecture.

The current study integrates neuroscience and architecture to explore how the human brain responds to multisensory stimuli (Arinta et al., 2024) in the workspace, focusing on how pandemic-related stimuli influence workers’ perceptions and experiences. By pandemic simulating, environments through virtual reality and *qEEG* technology, the study analyzes how individuals respond to frequent stimuli such as sound, light, and smell to understand how these stimuli impact spatial perception and workplace fatigue. This investigation lays the groundwork for Bayesian brain theory in the context of workspace design, revealing how the brain prioritizes relevant sensory modalities to manage cognitive load and adapt to changing environments. These findings enhance our understanding of multisensory integration in the workplace and offer valuable insights for designing more adaptive and supportive work environments in the post-pandemic era.

## 2 Theoretical framework

The influence of sensory stimuli in the workplace on cognitive performance has become an increasingly important topic, particularly in the wake of the COVID-19 pandemic. The pandemic fundamentally altered how people experience their surroundings, affecting the sensory inputs they receive in daily environments such as the workplace. For many, these changes have impacted their ability to perceive and interact with their workspace, introducing challenges to wellbeing and productivity (Suckley and Orel, 2024). In response to these challenges, researchers have emphasized the need to create multisensory environments that promote cognitive wellbeing and productivity while also taking into account the external and internal factors that shape an individual’s experience (Rajagopalan et al., 2022).

Human survival and daily functioning rely on the seamless integration of multiple sensory inputs, a process known as multisensory integration. This integration is vital for improving perception and reaction time, as well as for increasing accuracy in detecting stimuli. When visual, auditory, and tactile stimuli are combined, the brain is able to process information more quickly and with greater precision (Cornelio et al., 2020). Virtual reality technology has also been shown to improve performance and reduce cognitive workload when integrated into systems that provide multiple sensory inputs (Marucci et al., 2021). However, multisensory integration is not solely a tool for boosting performance in work settings. It also plays an adaptive role, particularly for individuals facing sensory impairments or aging-related decline. Researchers have found that this integration has therapeutic implications for conditions that affect the brain’s ability to adapt, reinforcing its importance across a range of contexts, from enhancing brain functions to supporting procedural learning (Meredith et al., 2012; Freiherr et al., 2013).

One of the key theoretical frameworks in understanding how multisensory stimuli are processed is the Bayesian integration theory. This theory, as outlined in the Handbook of Multisensory Processes, explains how the brain combines information from various sensory modalities using probabilistic rules to create a coherent perception of the world (Calvert et al., 2004). The brain weighs each sensory input based on its reliability and relevance in a given context. For example, visual and auditory inputs might be evaluated differently depending on the task at hand. The theory also emphasizes that multisensory integration is most effective when stimuli are presented at the same time and place, a condition known as spatial congruence. In work environments, this theory helps explain how sensory prioritization occurs, such as when visual input becomes more important than auditory cues in shaping spatial experiences. The brain constantly adjusts which sensory inputs to prioritize, depending on the environmental demands, and this study seeks to explore these dynamics in different workplace settings.

As workplaces adapt to these insights, there has been a growing interest in incorporating neuroarchitectural principles into workspace design. These principles are based on the idea that the environment plays a crucial role in shaping not only emotional and cognitive behavior but also physiological responses (Assem et al., 2023). Studies have shown that changes in brainwave patterns, such as beta, alpha, and circadian rhythms, can be linked to work fatigue, which in turn impacts cognitive performance (Wylie et al., 2017). Real-time monitoring of brain activity through EEG headsets has proven useful in tracking cognitive fatigue and preventing accidents in high-risk environments. Cognitive fatigue, often resulting from prolonged mental effort, has been linked to shifts in brain activity that gradually undermine performance, further highlighting the need for environments that mitigate these effects (Shamsul, 2021; Wang et al., 2021; van Doremalen et al., 2020; Haktanir et al., 2021).

The collaboration between neuroscience and architecture has been supported by theorists such as Pallasmaa, who argue that the experience of space is a deeply sensory process that involves all the senses, not just vision (Pallasmaa et al., 2017). By engaging multiple senses, people can experience a space more fully and deeply, enhancing their connection to the environment. This multisensory approach is particularly relevant in the design of workspaces, where the combination of sensory inputs can influence an individual’s emotional responses, productivity, and overall wellbeing (Anderton, 1991).

In conclusion, the literature on multisensory integration and workplace design reveals a growing understanding of how sensory stimuli affect cognitive performance. As the workplace continues to evolve in response to the challenges of the COVID-19 pandemic and beyond, incorporating these insights into design and management practices will be critical to creating environments that support both cognitive health and productivity.

## 3 Methods and materials

The experimental research model combines quantitative electroencephalography (*qEEG*) and virtual reality (VR) to explore how multisensory stimuli shape the subject’s brain experience in workspaces. The study focuses on three primary workspaces at Population Data Recording Places of Population and Civil Administration Offices (PCAO) (*Disdukcapil*) in Semarang (i.e., public service room, data recording room, and archive room) using the same sequence of stimuli. EEG data were collected using wireless *Emotiv Epoc X* 14-channel electrodes to detect brain wave activity. The electrode mounting system used in the device is the 10–20 system. Subjects explored the space with multisensory sensations for 20 s, and their brain responses were recorded using Emotiv Pro software. Following this, subjects answered questions about their feelings while working in the room, providing recognition data. The study measured subjects’ fatigue based on wave activity in 14 brain regions. The first analysis, the *t*-test, used average data on brain wave power bands in each response to stimuli to assess the stimulus effect on space. A comparison test was conducted between baseline (when opening and closing the eyes) and when given stimuli.

A second analysis, *repeated measure analysis of* var*iance (ANOVA)*, examined the stimulus effects on different brain parts and their waves. This analysis compared data between waves in each part of the brain, as seen in previous studies where EEG signals effectively detect fatigue (Zhang et al., 2017; Cortés et al., 2013). The *repeated measure ANOVA* results underscored *EEG*’s effectiveness in detecting fatigue (Hopstaken et al., 2016; Reyes-Muñoz et al., 2016). The psychophysiological mixed effect model in the analysis produced findings applicable to other studies (Bagiella et al., 2000). Power band raw data from Emotiv Pro software were used in this research for the second analysis, separated according to the timestamp when the subject responded to the stimuli.

The third analysis involved tabulating the subjects’ answers about spatial recognition influenced by the administration of test stimuli. This stage aimed to observe the effect of stimuli on the subject’s recognition of space, proved by significant brain activity. The *independent component analysis (ICA)* process was used on raw data according to standard procedures to facilitate the visualization of data processing results.

The process of interpreting conclusions involved combining the results of the analysis as the process of guiding perception to perception itself, including (1) *t*-test with *P- and T*-statistic values from Durbin-Conover, (2) *repeated measure ANOVA* with *P*- and *F-values*, and *estimated marginal mean* results, and (3) stimulus recognition answer results regarding space. The subject’s response to spatial recognition was linked to the four pillars of human beings in response to spatial conditions: physical, psychological, intellectual, and emotional (Assem et al., 2023). Hypothetically, this method could reveal how multisensory stimuli significantly influence brain activity for cognitive behavior in pandemic workspace stations. Holistic recognition that the remaining memory of workplace spatial experience during the pandemic was obtained from part responses and deep brain waves will create a fundamental workplace improvement in the post-pandemic era.

### 3.1 Stimuli variables

**Figure 1:**
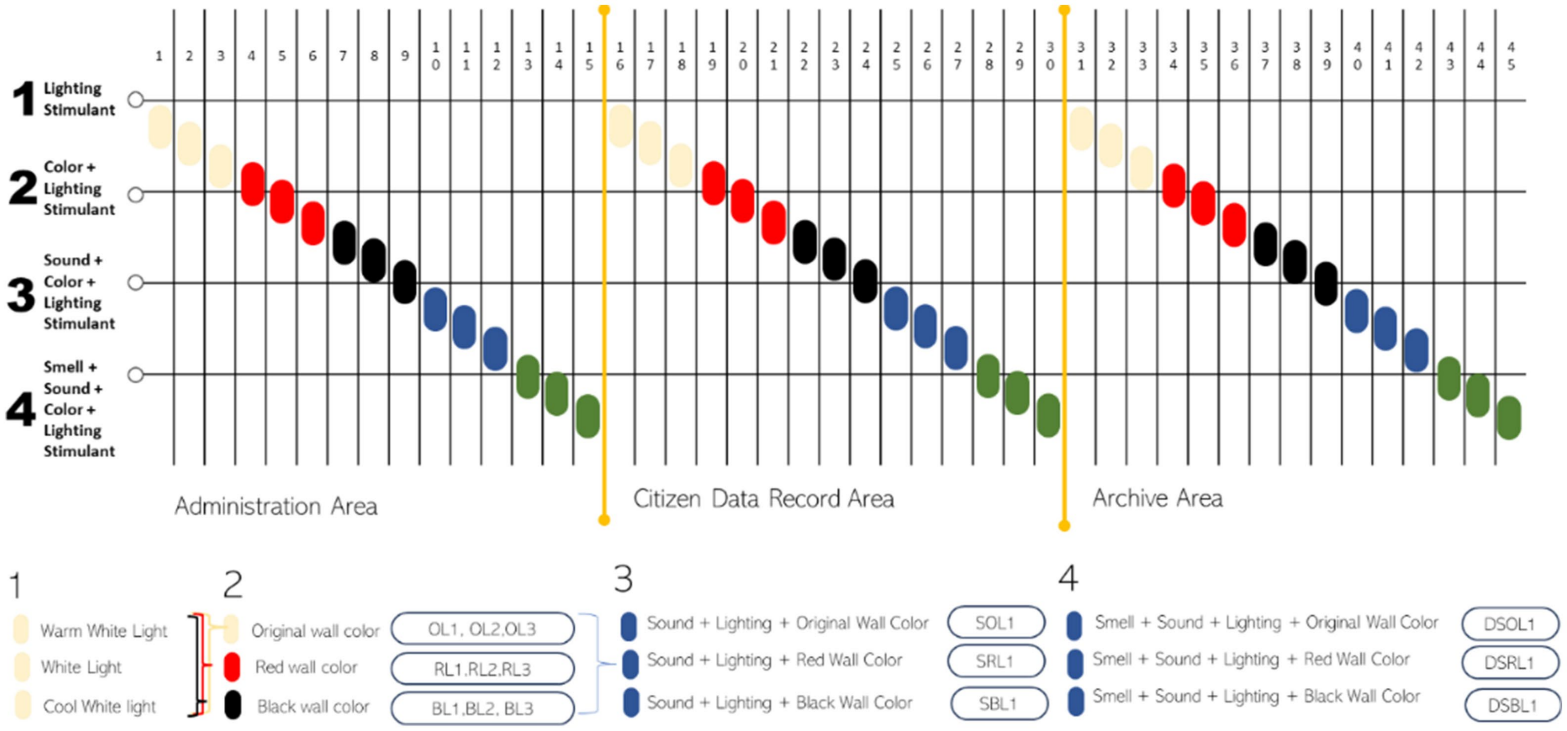
Procedures for adding stimuli during experiment (Author, 2021).

The exploration engages stimuli that interact with three senses simultaneously. The time required for data collection is approximately 90–100 min with a total of 45 variations of stimulants given. Thus, the stimulant observation period is carried out for 100 s after the stimulation is given for 20 s. Audience tested in pandemic fatigued stimulation relation on architectural studies like color and light, sound, and smell. Visual stimuli included versions in light (lamp) and coloration, with three forms of lamp versions (warm tones, everyday white tones, and tones toward blue) and three wall paint color versions (original (ivory white), red, and black) provided with VR. In this case, the brain’s BMI is not to interact directly with VR but only to observe. Audial stimuli included sneezing and coughing sounds and the smell of disinfectant. The observer used unequal factorial composition in three versions: visible, visual–audial, and visual–audial–olfactory, repeated in all three regions (Figure 1).

In this study, the stimulation of touch and taste was not tested. The room’s air temperature was maintained at 24°C at some point in the experiment, with a relative humidity of approximately 70%, adhering to thermal comfort standards in Indonesia. Taste changes into something not blanketed as no food or drink items within the workplace would cause a unique taste sensation. Auditory and olfactory stimuli had been the focus, with two versions: stimuli and no stimuli. The stimuli included the sound of sneezing or coughing and the smell of disinfectant, reflecting situations during the pandemic. Data collection concern*s quantitative EEG (qEEG)* records and qualitative data from the problem’s responses to questions after receiving the stimuli.

### 3.2 Object of study

**Figure 2:**
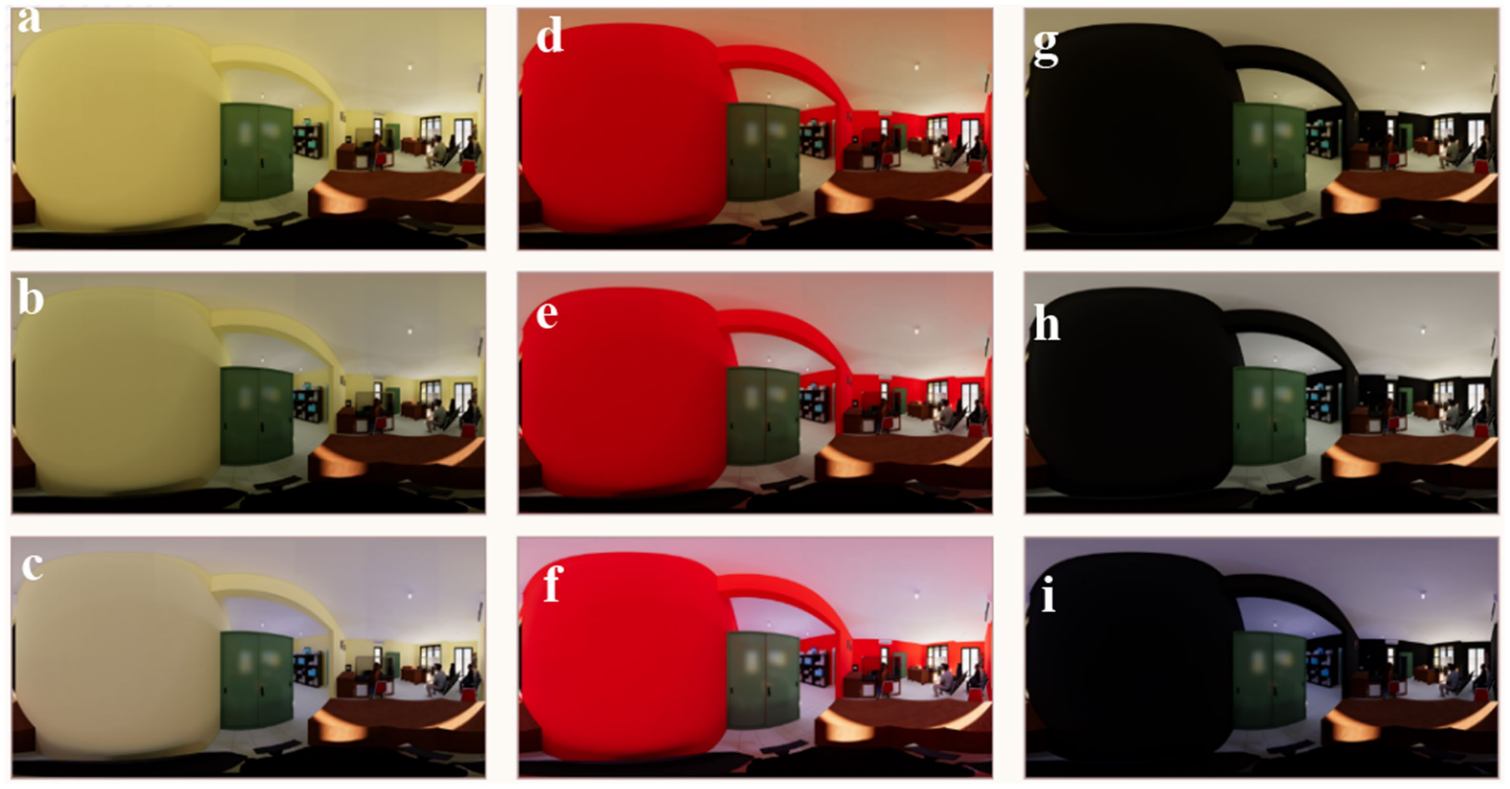
Visualization on VR **(A)** OL1–origin with warm white lamp, **(B)** OL2–origin with white lamp, **(C)** OL2–origin white lamp, **(D)** RL1–red with warm white lamp, **(E)** RL2–red with white lamp, **(F)** RL3–red with cool white lamp, **(G)** BL1–black with warm white lamp, **(H)** BL2–black with white lamp, **(I)** BL3–black with cool white lamp.

The research was carried out in Semarang’s four Population and Civil Administration Offices (PCAO). The PCAO is a district office, and every district has a Population Data Recording Place (PDRP). Semarang city has 16 PDRPs placed in each district. The four PDRP experimental places are (1) PDRP Gayamsari, (2) PDRP Semarang Barat, (3) PDRP Candisari, and (4) PDRP Tembalang. Each PDRP has three predominant work areas: (1) administration area (where residents explain the applications submitted, ID cards, family playing cards, and different certificates), (2) data record area (where citizens’ information, including images, biometrics, and fingerprints, is recorded), and (3) archive area (where the processed files are saved) (Figure 2).

Architecturally, the administration area, data recording room, and archive room interiors mirror the standard interior style of current country workplaces. Some workplaces have used guardrooms since the pandemic, with the data recording room next to the general public administrative place. Generally, the arrangement of those spaces emphasizes functional performance.

### 3.3 Participants

**Figure 3:**
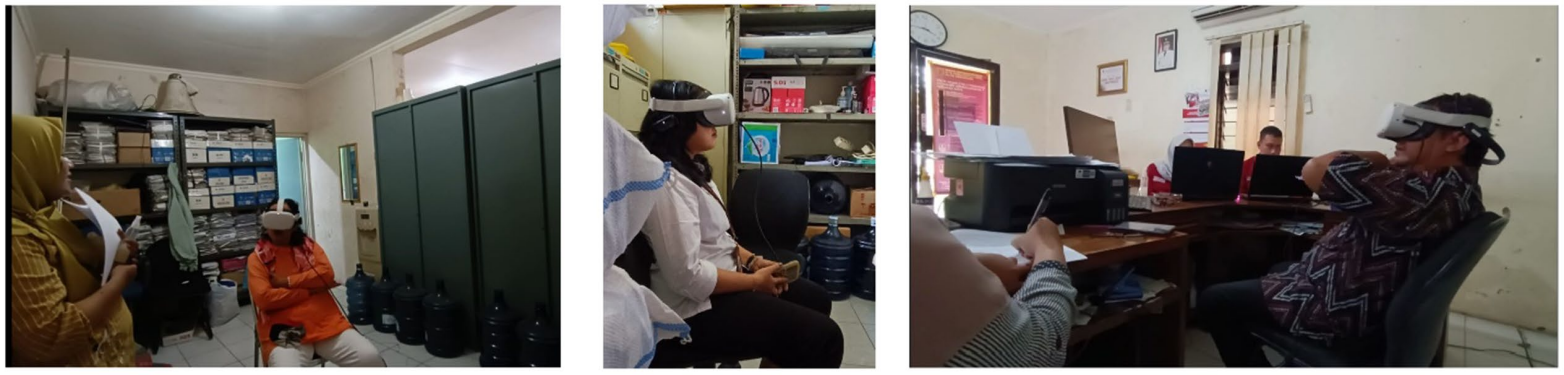
Data collection process at Disdukcapil Semarang office (Author, 2020).

The subjects of the study in Figure 3 were workers at PCAO Disdukcapil Office: (1) Gayamsari District (GY), two employees; (2) Candisari District (CS), two employees; (3) Tembalang District (TB), three employees; and (4) West Semarang District (SB), three employees.

**Figure 4:**
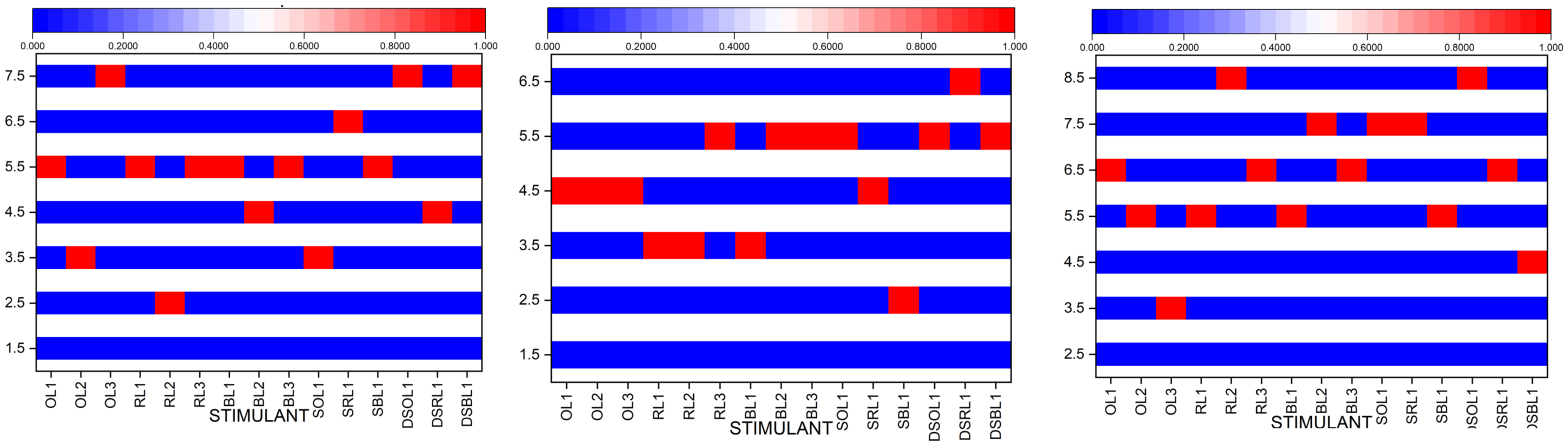
Stimuli that have a high degree of significance (Author, 2024).

The research sample was determined using proposal sampling. The criteria for this sample are related to permanent employment status (already having experience working at the PCAO for 3 years) and a minimum undergraduate education. Based on these criteria, 10 people worked in four types of PCAO Disdukcapil from 16 offices in Semarang City.

### 3.4 Procedures

This experiment combines two tools, EEG and VR Headset. A data retrieval stage consists of the following steps: (1) Record *baseline* conditions by opening and closing the subject’s eyes for 20 s using Emotiv Pro. (2) Provide an animated visualization video of the subject’s workplace 3D model. (3) Start adding visual stimulants of warm white (O.L1), white (O.L2), and cool white (O.L3) lights to the workspace with the original ivory white wall color. Observe the subject with visual exploration for 20 s, followed by asking questions about space. (4) Add red and black to the wall, combined with stimulant lights, warm white (R.L1 and B.L1), white (R.L2 and B.L2), and cool white (R.L3 and B.L3). Observe the subject by visual exploration for 20 s. (5) Introduce sneezing and coughing (S.O.L1), red (S.R.L1), and black (S.B.L1) sounds in rooms with ivory white, red, and black walls, along with varying light conditions. Observe the subject by visual exploration for 20 s. (6) Add a disinfectant smell that is applied around the subject accompanied by the sound of sneezing and coughing in rooms that have original colored walls (ivory white) (D.S.O.L1), red (D.S.R.L1), and black (D.S.B.L1)—20 s of stimulant exploration time followed by questioning. The experiment concluded with the final recording of EEG data. Following the administration of 15 different stimulus combinations in the administration room, the same procedure was applied to the data recording and archive rooms.

## 4 Results and discussion

**Figure 5:**
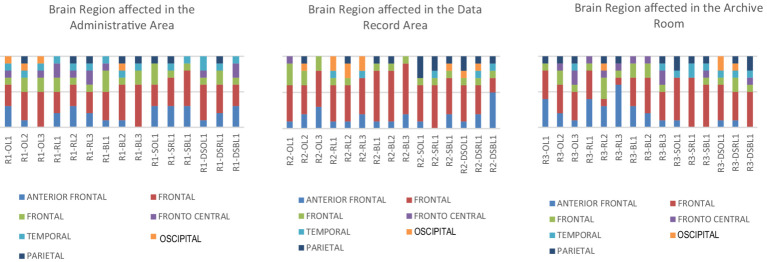
Brain significance responds to the multisensory in PCAO tested area (Author, 2024).

Initial analysis suggests an average distribution of data, which is confirmed by a *Shapiro–Wilk* probability lower than 0.001. The *t*-test can be a powerful method for performing parametric analysis of data. As shown in Figure 5, importance ranges can vary. This diagram illustrates the payoff across three rooms, the administration area, the data recording area, and the archive room attention on the red portions of the stimulus to understand the image.

Figure 4 shows the contrast of the responses to stimuli. Based on the statistical results, certain stimuli show a *p*-value of less than 0.001, which indicates high importance. The quantity of stimuli that subjects respond to can be visualized with a heatmap. The heatmap’s indicators on the left represent the number of subjects responding. The Y-axis indicates the percentage of participants who showed a critical response. The more significant the response is, the more influential the impact on the stimuli.

On The Public Administration area, the stimulus that was most commonly that was cool white light (O.L3) which (excellent white light variations), which has a mean value of 3.87, *T*-statistic number of 3.87, D.S.O.L1 with an average of 5.92, and D.S.O.L3 which has an average of 4.08 (olfactory stimulation variation was tested using black and original color in the presence of random light fluctuations). The recording room for data is a single significant stimulus; it is the D.S.R.L1 variation (smell and the sound of a room, a reddish color, and random light variations). The room for archive recording has two significant stimuli. The R.L2 variation has an average *T*-statistic score of 7.08 (reddish visual space with white neutral light) as well, and D.S.O.L1 has a *T*-statistics value of 4.93 (olfactory and sound visual space with the original hue (ivory white) and random variation in light). In the three areas tested, the six most significant stimulations were uncovered based on the results of the statistical payoff.

From the stimuli that have been tested statistically, it shows a significant response compared to baseline conditions. The graph (Figure 5) shows the spectral power distribution in the range of 0–25 Hz when responding to one of the six stimulants, the O.L.3 stimulant, with a focus on the effect of bluish light. Bluish light usually improves alertness and cognitive activity by reducing alpha waves (8–12 Hz) and increasing beta or gamma. However, if the data show stability at a frequency of 10–20 Hz with no significant change, this indicates that bluish light exposure may not be enough to stimulate significant increases in alertness or brain activity or that there are other factors affecting brain responses.

**Figure 6:**
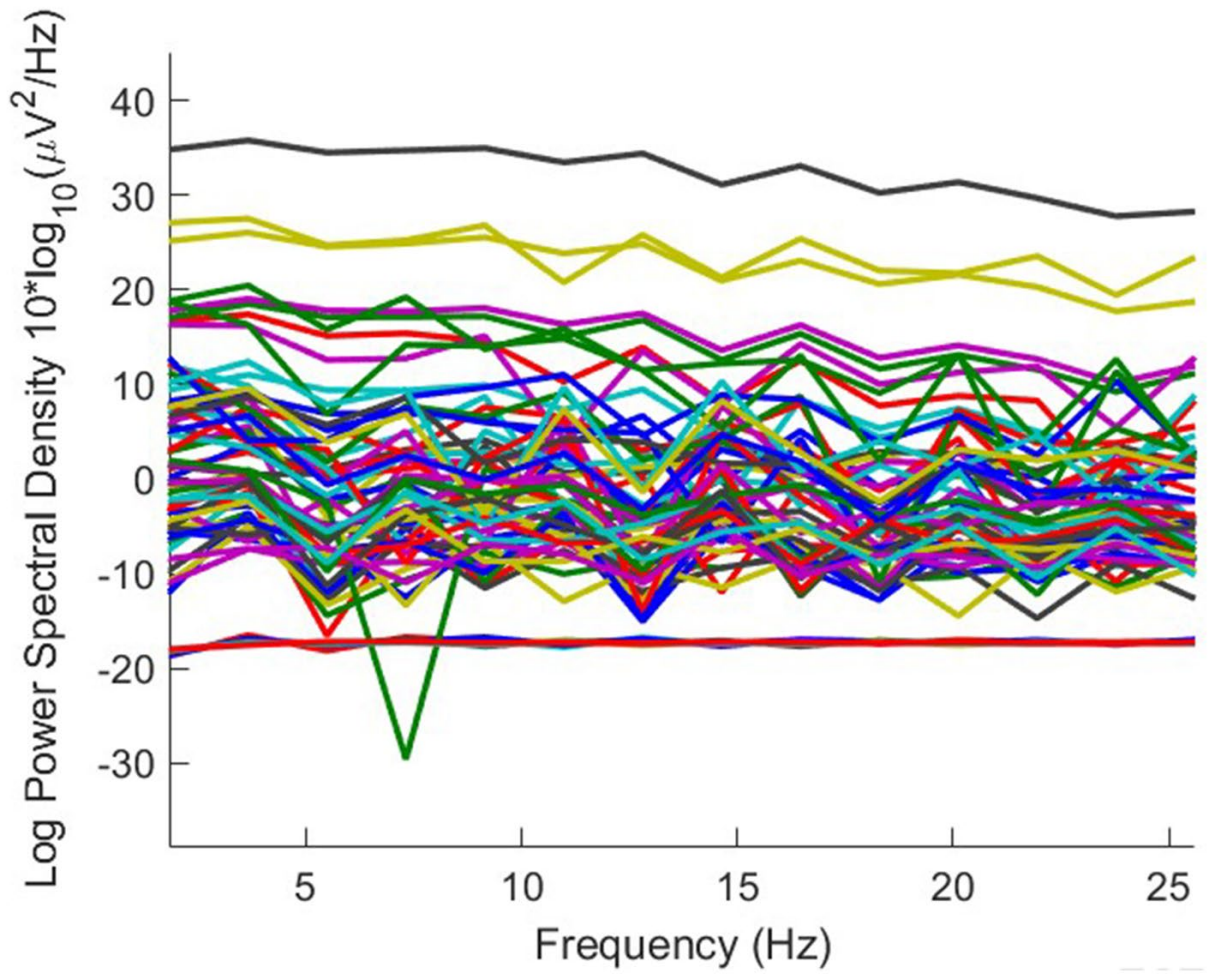
Visualization of brain activity at O.L.3. Stimulant in all wave classification of 14 channel (Author, 2024).

The multisensory stimuli brain performance demonstrated by the statistics outcome in Figure 6 explains individual dynamics reacting to stimulation across different brain regions. The outcome reveals dominant theta activity accompanied by *beta, alpha, and gamma* waves. It is a diverse result; based on the data, specific vital outcomes are as follows: (1) some individuals respond calmly to stimulation, as evidenced by more excellent stability in brain activity in particular areas. The same can be seen in the lowest *F*-value of 4.69. In contrast, the subjects showed more activity with higher brainwave activity, registering it, which resulted in an *F*-value of 278.942. (2) The changes in activity between three rooms, the administration area, data recording room, and archive room, were insignificant. Activity in the frontal brains generally resembled. Activity in the central frontal region was most prominent in response to the administrative region’s visual stimuli (light). Similar patterns were observed within the data recording room. However, a distinct pattern was observed when the brain responded to light in the archive room.

Alongside the study shown in the figure above, comparing the brain’s area responses with the *Post-hoc* test (with *Ptukey, Pscheffe, and Pbonferroni* results) produced an outcome near 1.000. These outcomes indicate insignificant differences among the brain’s area, waves, and regions that respond to multiple sensory stimuli. In addition, even though brain responses to multisensory stimuli varied, there were no statistically significant distinctions when studied across regions, areas, and regions. This research has important implications for understanding the specific impact of multisensory stimuli on brain performance and cognitive processing.

The frontal brain (including the anterior frontal, front central, left, and right frontal) had the highest brain activity if stimuli were introduced. In the case of visual stimuli, particularly cool white light was used; statistical analyses found that the cool white light caused the most significant effect on brain activity in the frontal region, which comprises the prefrontal, supplementary motor, premotor, and the primary cortices of the motor. Cool white light produced higher *F*-values than different lighting options. The addition of color caused more significant cerebral responses over black and unique shades, highlighting the frontal lobe’s crucial function for processing visual information. This research which is in line with earlier studies (Talsma et al., 2006; Tang et al., 2016) shows the role of the frontal lobe in the integration of multisensory data to provide an immersive perception. However, when contrast was decreased, the activity of the occipital region (yellow) diminished, shifting activities to temporal and parietal areas.

Additionally, the addition of sound impacted the perception of visual information, resulting in significant *F*-values that indicate the dominant role of sound on perception and focus. The synchrony of visual, auditory, and olfactory stimuli showed complex multisensory integration. Statistical analysis revealed that the brain’s activity shifts to the temporal and frontal regions and increased response in the parietal region when smelly stimuli were added. In particular, the introduction of smell stimulated occipital activation in the structure and data areas, which increased activity in the parietal lobe and reduced activity in the frontal cortex. The interaction between frontal, parietal, and temporal cortices promotes the coherence of perceptual experiences (Freiherr et al., 2013; Ortiz-Terán et al., 2017). The ability of the frontal brain to multiple sensory stimuli reveals the versatility and complexity of neural mechanisms that allow for sensory integration (Moran et al., 2020; Radstake et al., 2023).

**Figure 7:**
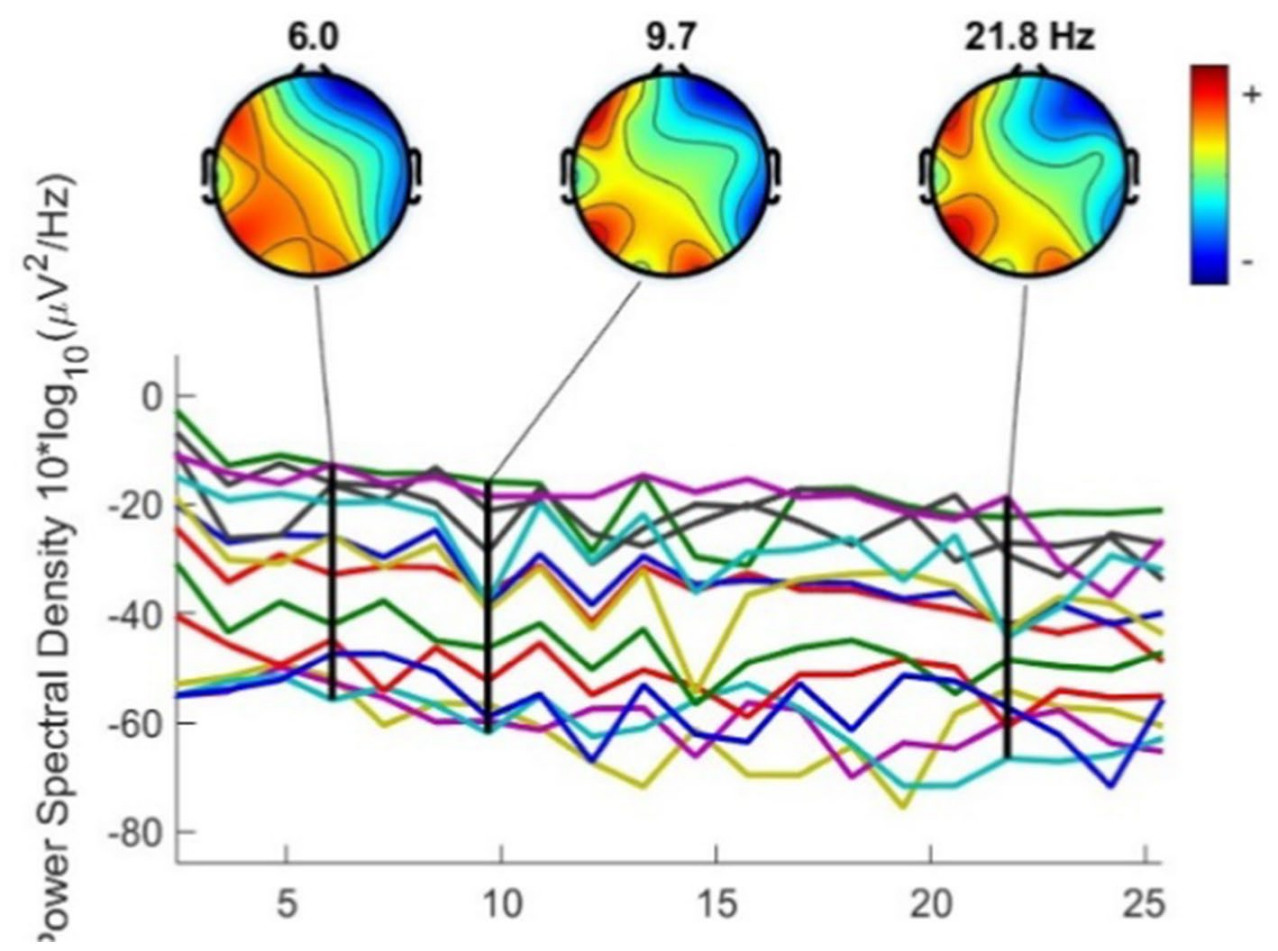
Visualization of spectral maps of brain wave activity at baseline conditions (Author, 2024).

**Figure 8:**
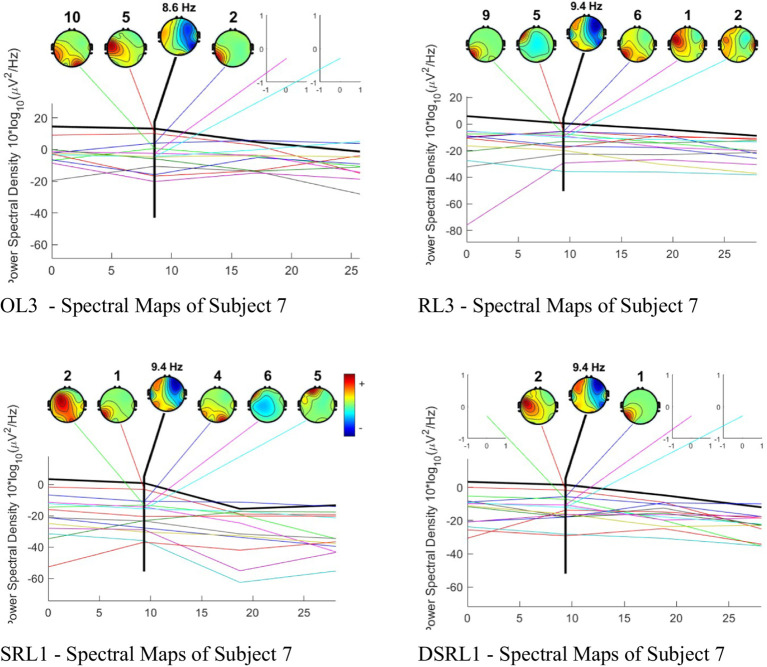
Spectral map visualization as stimuli are administered and added to each other (Author, 2024).

The next step is to visualize ICA to enhance the statistical outcome in Figure 7. This method separates artifacts and the database and determines when the stimulus has been introduced. ICA is used to see the actual performance of brain waves, by separating the motion wave artifacts from eye movement and facial muscle activities. The detection of the subject’s attention to the stimulant will be clearly observed. After filtering with EEGLAB software, the brain activity alerts can be seen in the following figure. The evaluation of spectral maps illustrated in Figure 8 shows the situation at baseline, including artifacts in theta waves (4–7 Hz), the alpha wave (8–12 Hz), and the high beta wave (18–30 Hz).

**Figure 9:**
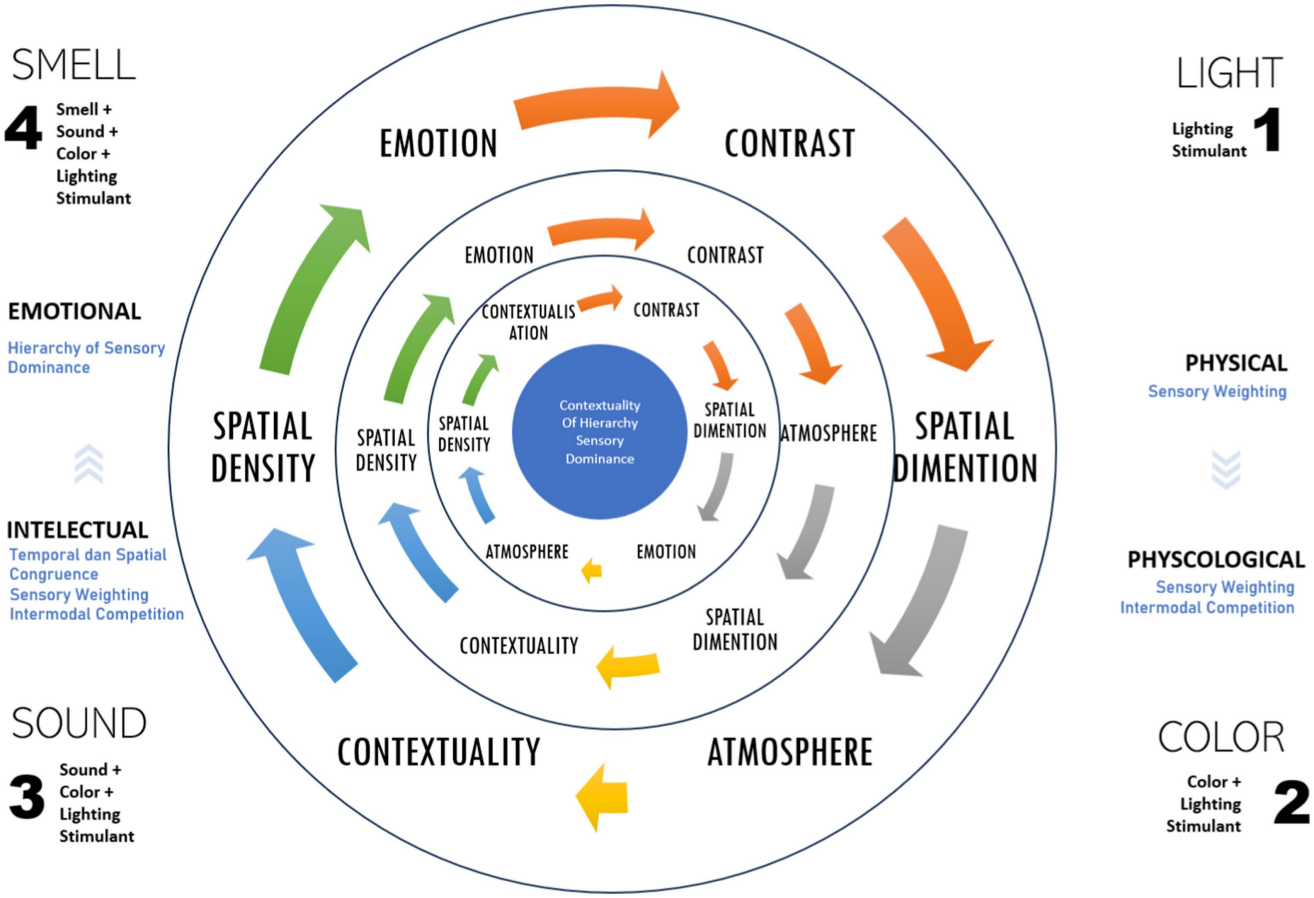
Interrelation between spaces. Stimuli and brain responses (Author, 2024).

The spectral map of Figure 8 illustrates the subject’s responses to the cold white light fixture (Figure 9—the OL3) that triggers the alpha wave with a frequency of 8.6 Hz. The presence of three different artifacts followed this. If the version for lighting fixtures was paired with color, alpha wave activity increased to 9.4 Hz with five distinct artifacts. Sound and smell enhanced the influence of alpha waves while maintaining the same rate at 9.4 Hz. The effect of transitions on the spectral map is interesting, mainly due to the addition of red color and sound stimuli.

Theta waves are typically linked with memory processing and drowsiness. Alpha waves, triggered during relaxation, and the high beta waves, which can be associated with intense cognition, are caused by muscle contractions as well as the electrical activity of the environment. These interferences can affect the understanding of signals from EEG. Preprocessing methods, including independent component analysis (ICA) and filtering, ensure that the signals analyzed accurately represent brain activity. Finding and eliminating artifacts is crucial to ensure the information authenticity and verify findings from the study. Ultimately, the results have been meticulously processed to reproduce the brain’s activity under the influence of impactful stimuli.

In addition, artifacts must be identified to reveal visual stimuli interactions with hang hues. Sneezing and coughing disturbed subjects, triggering more efforts as part of the brain’s adaption system. The alpha frequency in the EEG signal is a key indicator that can be used to assess mental (Tran et al., 2020) and cognitive fatigue (Trejo et al., 2015). A low level of alpha activity, less than 13 Hz, is a sign of less brain activity. The boost in alpha waves observed during the response to visual stimuli is a crucial clue into how strong stimuli impact cognitive conditions, specifically during the pandemic of fatigue. The fact that the intensity of the alpha waves is subtle and does not fluctuate dramatically in the presence of colors, sounds, or smells indicates that the brain can recognize and integrate these sensory stimuli without being overstimulated. If color is added to smell and sound, the frequency stays constant, suggesting that these stimuli have been taken energetically. The addition of these stimuli affects the cognitive load and overall vigilance. They certainly affect the brainwave frequency balance, which includes alpha waves, which can be linked to fatigue (Liu and Duricka, 2022). Stress at work is also connected with a person’s state of mind and cognitive workload, workflow, work-related struggles, and the climate of an organization (Arinta et al., 2022; Arinta et al., 2023). This weak alpha exercise identifies employees’ fatigue levels (Roy et al., 2013).

Based on the perception responses from the three tested rooms at PCAO Disdukcapil, Semarang, six key elements were identified as critical in workplace design: contrast, dimension, atmosphere, contextuality, density, and emotion (Figure 9). These elements were mapped across the different workspaces, with the outermost circle representing perception results from the public service area, the middle circle reflecting the data recording area, and the innermost circle corresponding to the archive area. These perceptual responses were further correlated with brain performance (Figure 8), particularly in areas such as the frontal cortex (responsible for cognitive and perceptual processing), the occipital lobe (associated with visual responses), and the temporal lobe (processing smell and color stimuli). In all three workspaces, multisensory stimuli—such as sound, light, and smell—triggered intermodal competition, where different sensory modalities competed for dominance in brain processing.

Among the various lighting conditions tested, cool white lighting produced the most significant response, statistically associated with greater mental relaxation and comfort due to its impact on contrast and atmosphere. Dynamic comparisons of brain performance in response to these lighting variations, based on previous independent component analysis (ICA), revealed that cool white lighting triggered alpha wave activity at 8.6 Hz. A decrease in brain activity in the frontal region indicated reduced cognitive processing demands, suggesting that the brain shifted toward more automated sensory processing. Bluish lighting, combined with good spatial contrast, was processed more efficiently, as reflected by stable sensory weighting in the occipital lobe, reducing cognitive load and enhancing decision-making, visual comfort, and relaxation.

Introducing red area and the variation of light stimulation across the various lighting conditions cool, warm, and standard white in contrast, it raised brain activity to 9.4 Hz. This heightened activity was particularly focused on the visual modality in the data recording area and on spatial perception in both the administration and archive areas. Red paint significantly influenced visual processing, creating a more pronounced atmosphere in the data recording room. On the other hand, black area and the variation of light stimulation with the same variations reduced brain activity to 9.1 Hz, particularly in the frontal regions. In the administration area, the black area within light stimulation influenced the atmosphere, while in the data recording room, it shifted perception toward spatial dimensions. In the archive room, black area and the variation of light stimulation had a stronger impact on workers’ emotions. Through sensory weighting and intermodal competition, the brain was able to prioritize the most relevant sensory modalities, thereby enhancing productivity and comfort in each workspace. Red area attracted more visual attention, sharpening spatial awareness and supporting decision-making and navigation. Meanwhile, black paint stimulation facilitated emotional and atmospheric adaptation, allowing workers to balance their focus and emotional state, particularly in quieter spaces such as the archive, where spatial dimensions are less significant and emotions play a larger role.

Furthermore, the introduction of sound and smell strengthened the dominance of alpha waves, maintaining a steady frequency of 9.4 Hz. Frontal brain activity fluctuated when these stimuli were combined with red area and the variation of light stimulation, while temporal activity increased with the addition of new stimuli. Sound shaped contextual awareness, influencing perception in the administration and data recording areas by defining the specific ambiance of each workspace. In the data recording area, sound contributed to a distinctive atmosphere. Meanwhile, smell influenced the perception of spatial density in work environments, where higher occupancy levels made smells more prominent, guiding workers’ emotional responses. The interplay of sound and smell illustrates how individuals adapt to their surroundings through intermodal competition, where the brain actively selects the most relevant stimuli for processing. Sound proved more dominant in cognitively demanding spaces such as the administration and data recording areas, while smell played a more significant role in denser environments such as the archive, affecting emotional and spatial perceptions. Sensory weighting ensured that the brain prioritized the most relevant modality based on specific workspace needs, helping workers navigate and respond to their environment effectively.

Allowing workers to quickly identify the source of stimuli and link it to relevant tasks or spatial conditions, temporal congruence further ensured that sound and smell were seamlessly integrated within the workspace. This congruence enabled the brain to maintain stable perceptions despite varying stimuli, reinforcing concentration and minimizing distractions. The hierarchy of sensory dominance determined which modality the brain prioritized, allowing workers to remain focused, avoid distractions, and make quick decisions when necessary. As a result, these multisensory stimuli supported the brain’s ability focusing on the most relevant sensory inputs, ultimately enhancing productivity and comfort.

Recent research suggests that the brain weighs sensory modalities based on their relevance to specific workspace situations. Multisensory stimuli significantly impact human behavior and decision-making strategies (Auvray and Spence, 2008). Previous studies also link multisensory integration in brain performance with Bayesian (Calvert et al., 2004). The brain continually processes new information, updating perception through sensory weighting and selecting the most helpful modalities for individuals to navigate and adapt to their workspace (Ernst and Bülthoff, 2004). In Bayesian theory, the brain acts as a probabilistic machine, constantly updating its hypotheses about the world based on received sensory evidence, which is well-suited to explain the multisensory integration process occurring in workspaces (Angelaki et al., 2009). This study reinforces the Bayesian brain concept to enhance our understanding of perception and survival mechanisms in the workplace. Focus to this development are the concepts of sensory weighting and modal dominance. Sensory weighting refers to the brain’s ability to determine which sensory modality is most relevant based on the conditions of the workspace. For instance, sound is more dominant in administration areas because of its association with cognitive tasks such as communication, while smell plays a greater role in archive spaces due to its emotional and spatial effects. On the other hand, intermodal dominance determines which sensory modality has the greatest influence on perception in a specific environment.

The brain continuously updates its sensory perceptions through the Bayesian brain mechanism, adjusting its processing of stimuli and prioritizing the most relevant modality for each workspace context. For example, in an administration area where verbal interaction is prominent, the brain assigns higher sensory weighting to sound because it is crucial for communication and cognitive tasks. By intensifying the processing of sound, the brain provides essential context for understanding the work environment and supports quick, accurate decision-making.

Conversely, in the archive, the brain shifts its priority to the olfactory modality with distinctive smells, which triggers emotional and density-related responses. Strong smells in confined spaces elicit emotional awareness, making workers more conscious of their surroundings. In areas featuring striking colors such as red, the brain focuses on visual modalities, particularly for navigation and spatial comprehension—an essential aspect in data recording and administration areas. These examples illustrate how the brain dynamically adjusts to the sensory demands of different work environments, ensuring optimal perception and decision-making.

## 5 Conclusion

Multisensory integration in the workplace plays a critical role in shaping employee perception, influencing both productivity and comfort. From an architectural perspective, this study highlights how visual elements such as light and color significantly affect the perception of spatial dimensions and overall ambiance. For instance, red area and the variation of light stimulation enhance spatial awareness, while black-colored areas create a more emotionally charged atmosphere. In denser spaces such as archives, olfactory stimuli, particularly smell, become more influential, impacting spatial perception and emotional responses among workers.

From a neuroscientific standpoint, these findings align with the Bayesian brain theory, which posits that the brain continuously updates its sensory perceptions through sensorimotor weighting, prioritizing the most relevant sensory inputs based on the task at hand. The brain adjusts its focus to specific sensory modalities, such as sound in office environments and smell in archive spaces, to reduce cognitive stress and optimize performance. This dynamic adjustment ensures that the brain responds effectively to varying work environments, maintaining high levels of productivity and comfort.

It also comes with some issues. The tests were conducted with only three kinds of workplaces, with 10 participants, each of whom displayed distinct multisensory features. Furthermore, particular differences in the sensory response were not explored fully and could limit the generalization of the results. It is suggested that more diverse workspaces and work conditions be studied while including more sensory factors such as temperature and texture to learn how different sensory factors affect perception. Future research should consider the individual variations in how people respond to sensory stimuli and responses, such as factors like the person’s age and cultural background, which influence how workers respond to their environment.

## Data Availability

The raw data supporting the conclusions of this article will be made available by the authors, without undue reservation.
