# Supplementary material for: Brain spatial reconciliation through multisensory integration in the impact of pandemic fatigue on workplace
Source: Front Hum Neurosci. 2024 Dec 6;18:1419889. doi: 10.3389/fnhum.2024.1419889 (PMC11659956; doi:10.3389/fnhum.2024.1419889)
Supplement: Supplementary file 1 [file Data_Sheet_1.DOCX]

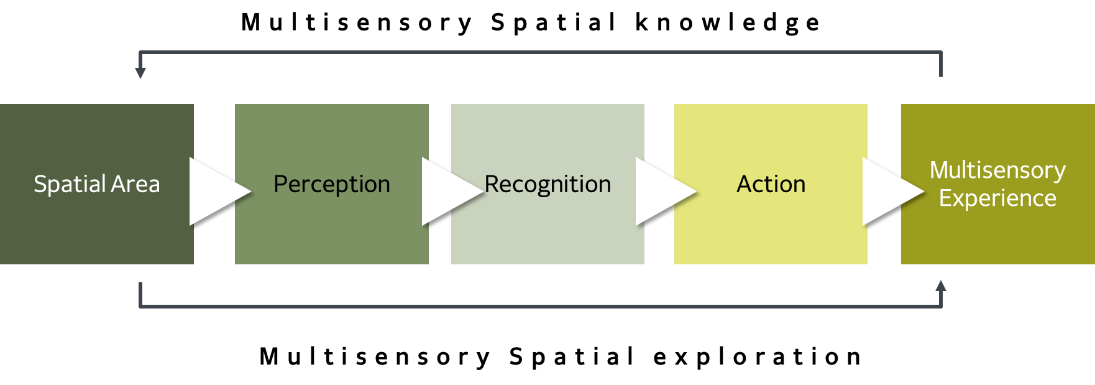


**Figure 1**. The formation of perception according to neuroscience^[[1]](#footnote-1)^.

**Step 1**

**Step 2**

**Step 3**

**Step 4**

**Figure 2.** Prosedur Analisa Data


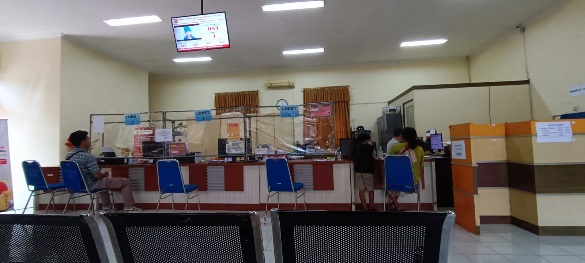

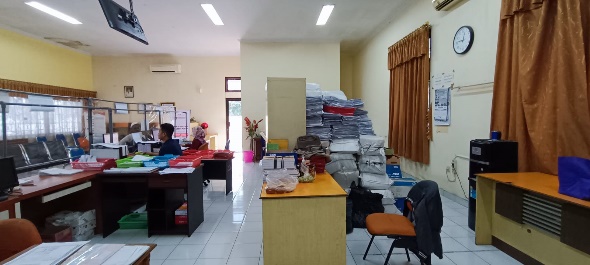


**Figure 3. Condition existing Kantor Disdukcapil.**

Tabel 1 Variabel dan variasi treatment dalam pengambilan data

| **Indikator** | **Parameter** | **Variasi** | **Komposisi Stimulan** | **Combination of Stimulant** |
| --- | --- | --- | --- | --- |
| Visual | Lights | warm white (L1), white (L2), cool white (L3). | All Given | O.L1, O.L2, O.L3, R.L1, R.L2, R.L3, B.L1, B.L2, B.L3. |
|  | Color | Original (O), Red (R), dan Black (B) |  |  |
| Audial | With/without Sound | Sneeze (S) | Audials are randomly selected one + visual representative of color variations + lamp variations. | S.O.L1, S.R.L1, S.B.L1 |
|  |  | Cough (C) |  |  |
| Olfactoral | With/without Smell | Disinfectant (D) | Olfactory given all + Audials Randomly selected one + visual representative of color variation + lamp variation. | D.S.O.L1, D.S.R.L1, D.S.B.L1 |


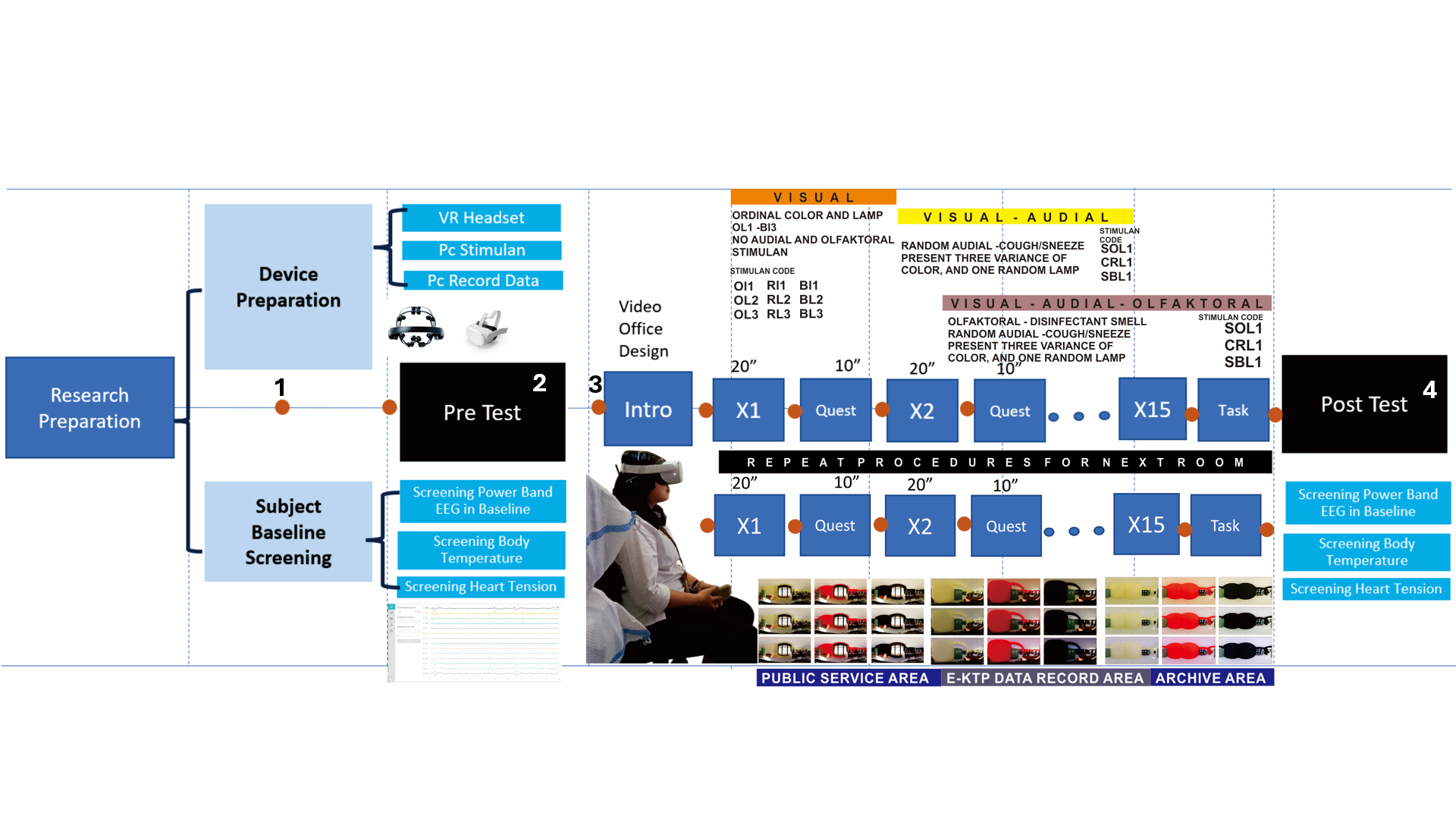


**Figure 4.** Data Records Procedure


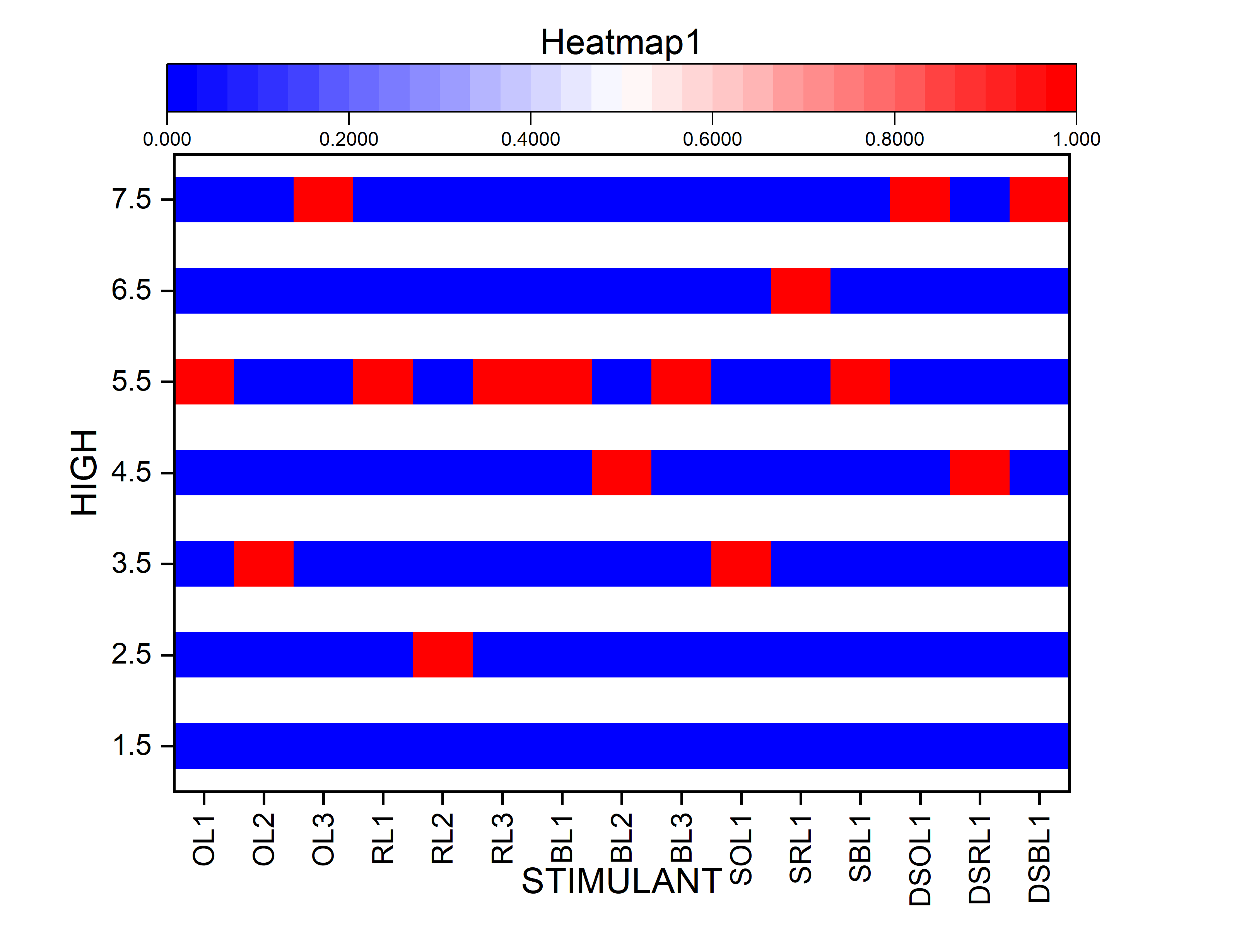

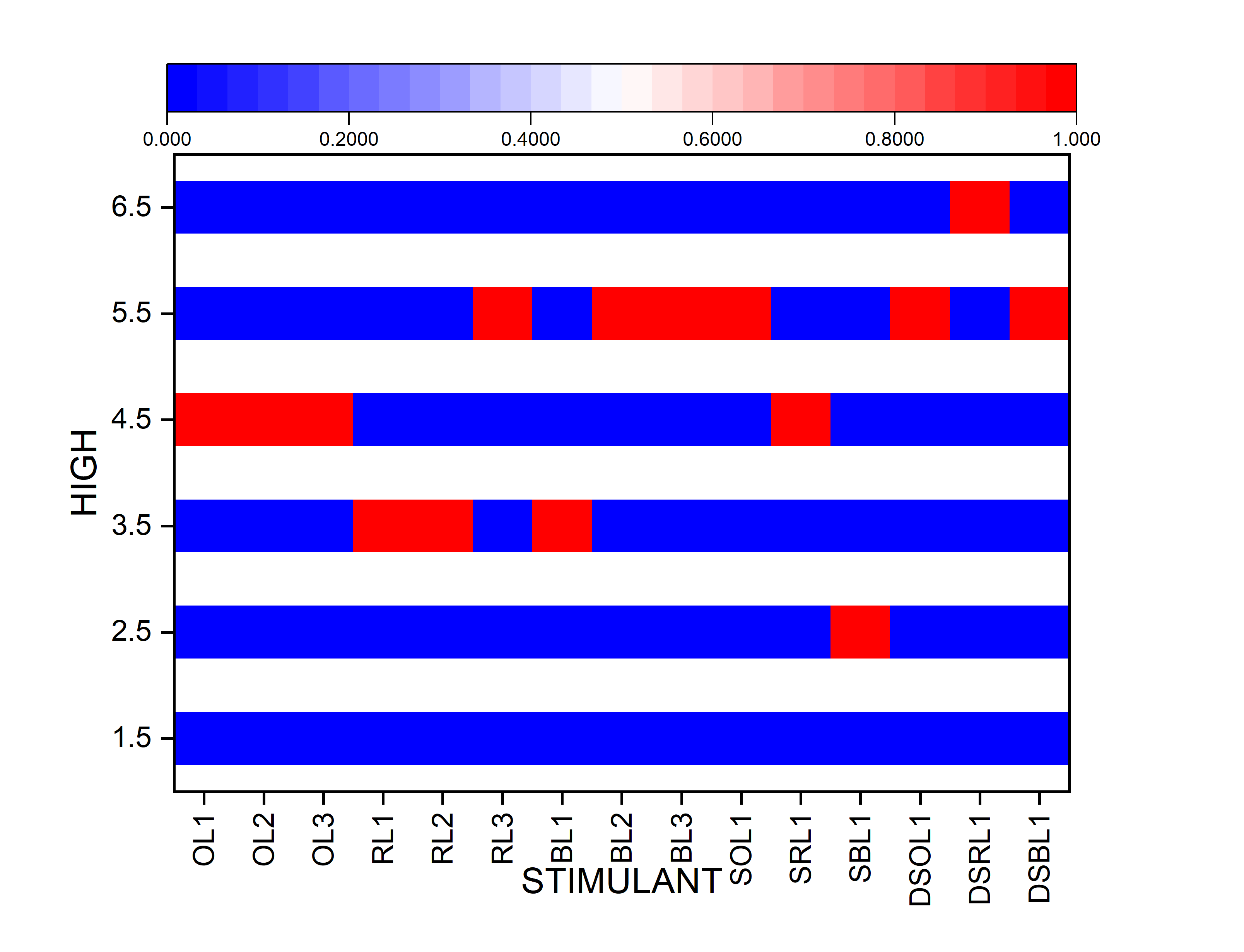

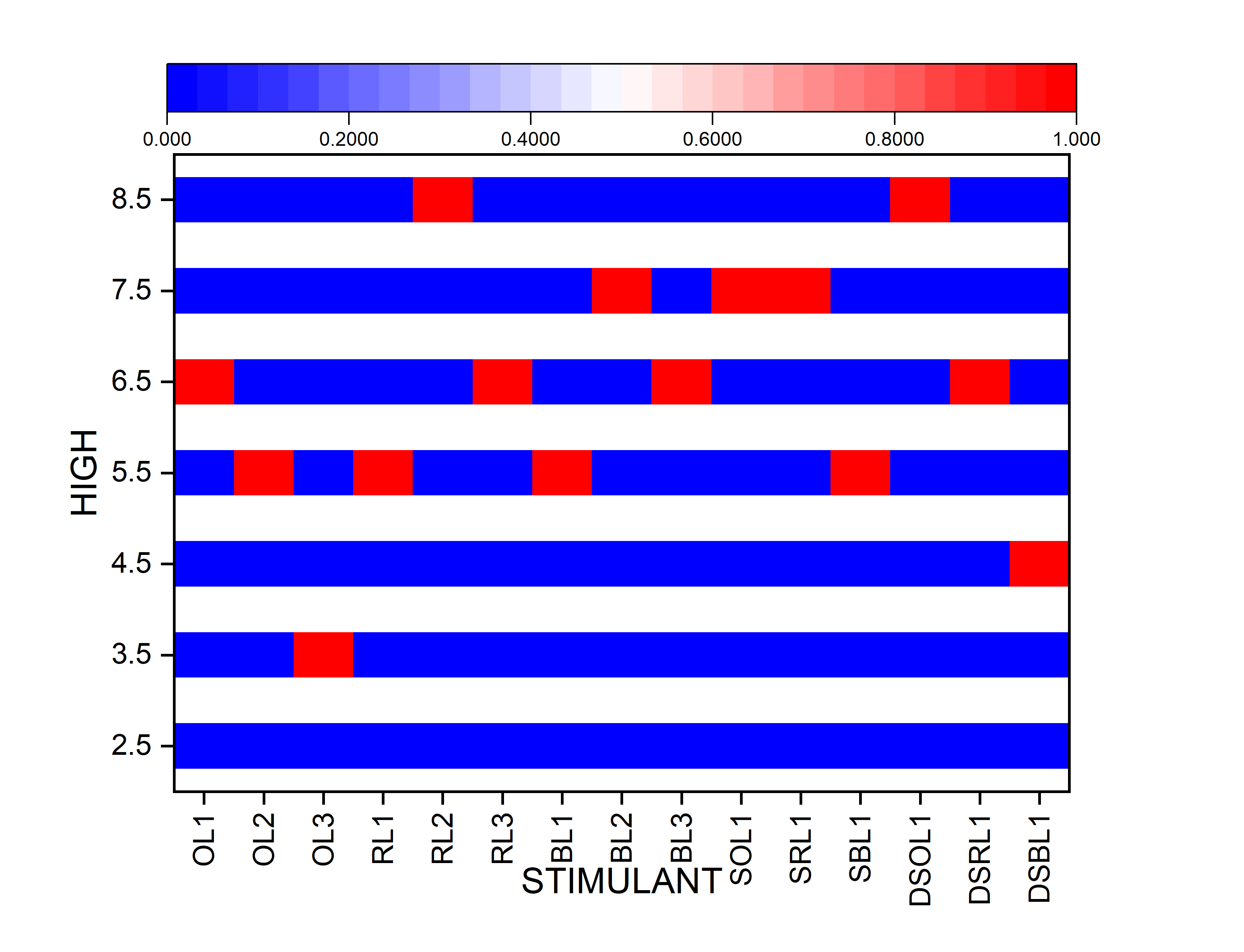


**Figure 5.** Stimulus that has a high degree of significance.


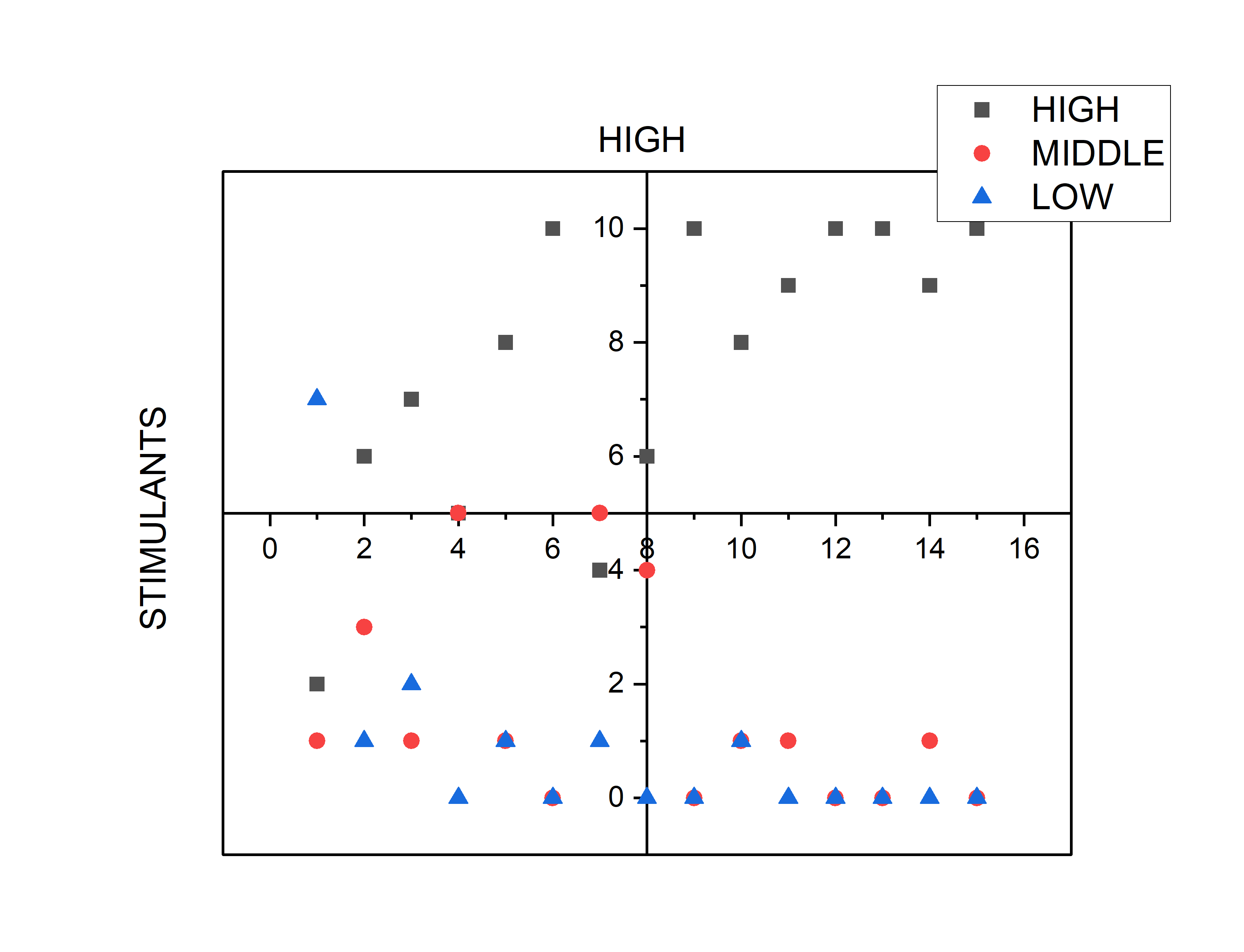

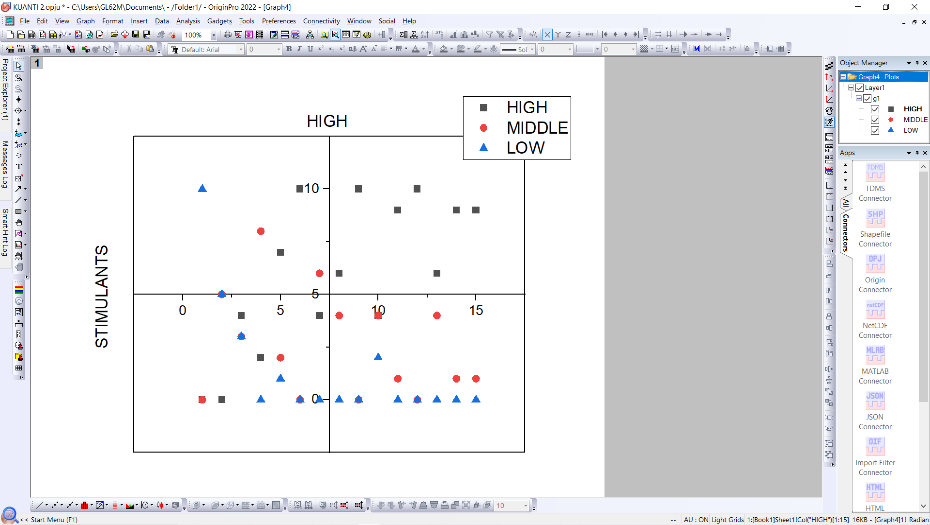

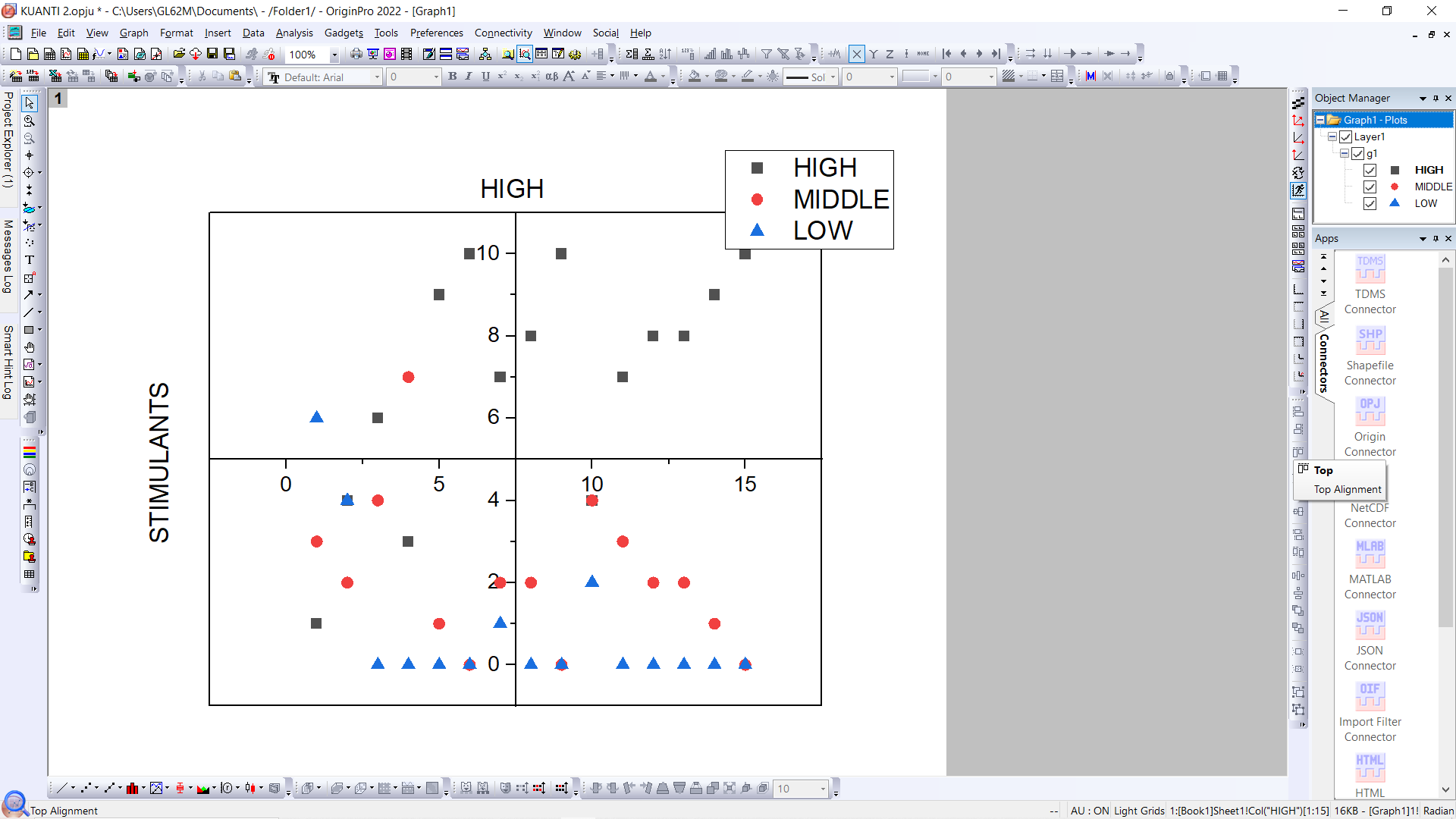


**Figure 6**. The process of formation of perception through the introduction of stimulants


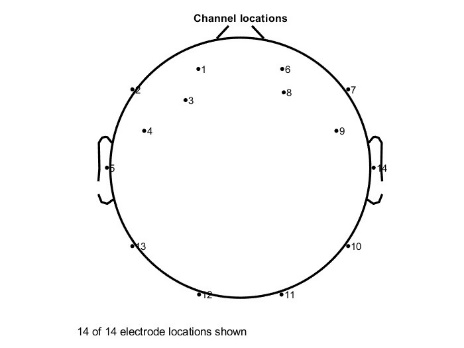

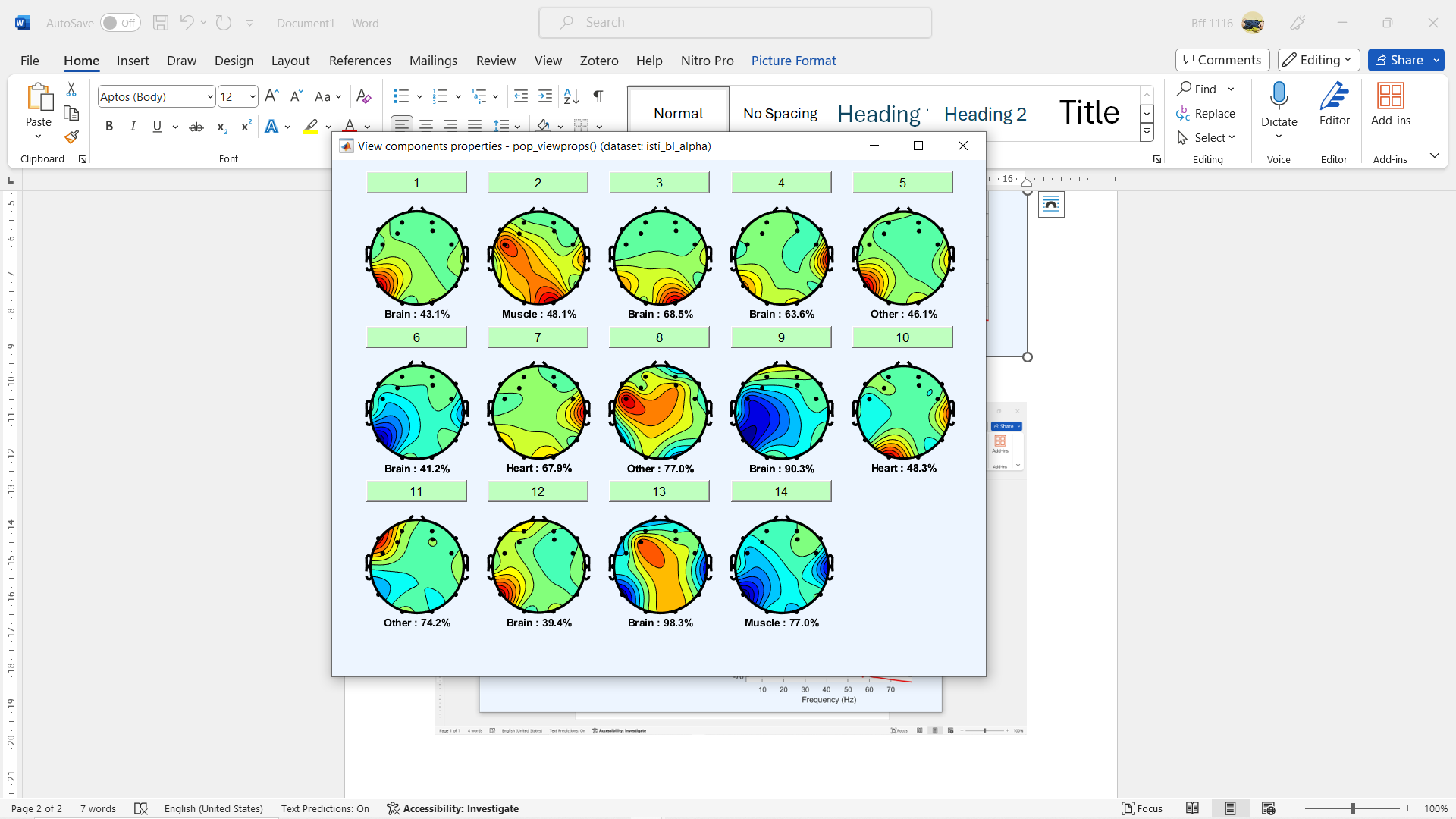

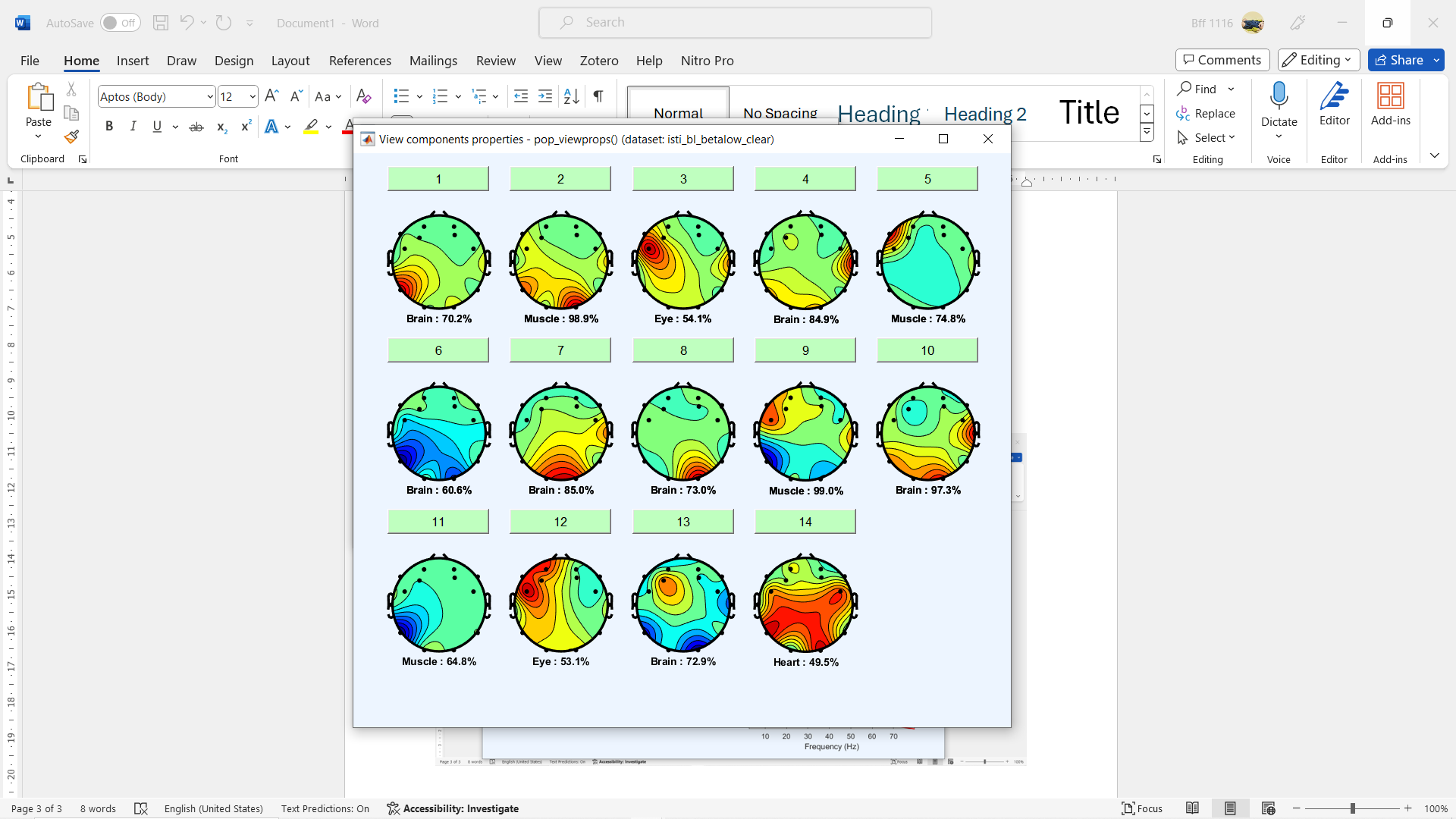


**Figure 7**. Visualization of wave activity, alpha-α, beta-β before stimulation


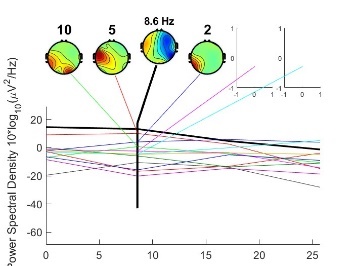

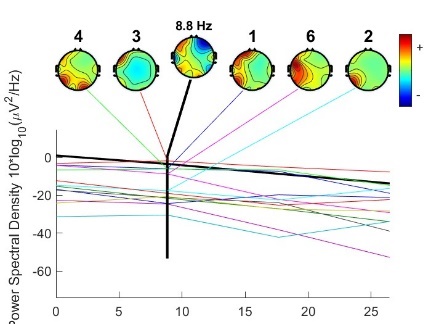

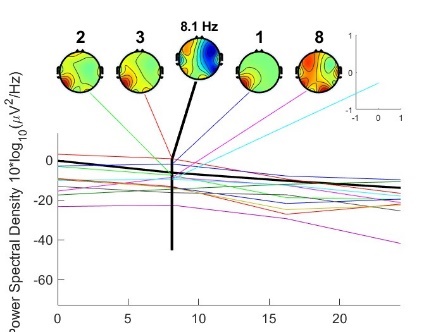


**Figure 8**. Visualization of beta waves on frontal and temproral sections

1. Goldstein and Cacciamani, *Sensation and Perception*. [↑](#footnote-ref-1)
